# Dr. Rigby

**Published:** 1822-02

**Authors:** 


### Medical Necrology and Biography.

IN one of our late Numbers, the melancholy duty devolved on us of announcing the death of Dr. RIGBY, the eminent surgeon and practitioner of midwifery at Norwich. Contemporary Journals recorded, nearly at the same time, the sudden demise of another distinguished surgical character, Mr. JOHN RING. Anxious to pay a just tribute to the memory of both these excellent men, we have collected a few particulars respecting the events of their lives, which we submit to our readers, in the full confidence that they must ever welcome every information which tends to rescue from oblivion the names of respectable members of the profession.

### DR. RIGBY.

Edward Rigby was born at Chowbent, in Lancashire, on the 27th of December, 1747, and was fortunate in being placed early under the care of Dr. Priestley, from whose example he derived that love of philosophical research, which formed one of the leading characteristics of his powerful mind. At the age of fourteen, he came to Norwich, and was apprenticed to Mr. Martineau, an eminent surgeon. At the expiration of his term of indenture, he completed, in London, the customary course of a medical education; and returned to Norwich, to exercise his profession.

While yet a very young man, his observation was strongly drawn towards some extraordinary facts that occurred in his midwifery practice, and he printed a treatise on the Uterine Hemorrhage; a book which excited considerable attention, and, though condemned by Dr. Denman, then at the head of that particular branch of science, has stood the test of time,—has passed through five editions,—has been translated into French and German,—and is now in universal estimation amongst medical men.

Two of Mr. Rigby's publications were, "A Plan for the Charitable Inoculation of the Poor," and "An Essay on the Red Peruvian Bark." One of his favourite objects, because one of the most important to human life, was the prevention of the ravages of that dreadful malady, the small-pox. In 1785, he printed a pamphlet, in which he started a very ingenious theory on Animal Heat, and applied it to the treatment of cutaneous disorders, inflammations, and some other diseases. This work exhibits strong marks of the author's ability and medical judgment; for he therein recommends the application of refrigerants to the skin in inflammatory diseases; and the present doctrine of cold effusion, as introduced by Dr. Currie, differs little from the treatment proposed by Mr. Rigby.

In 1788, he broke, for a short time, from his numerous engagements, and visited France, a part of Italy, and Switzerland, in company with W. Morgan, esq. the celebrated calculator, and actuary of the Equitable Office, and another friend. It so happened that they reached Paris just at the explosion of the Revolution. He was detained in that city a week, during which the attack of the Bastile, and the massacre of the Tuilleries, took place. He became acquainted with Turgot, Roland, and other persons distinguished at that period; and he has written a very animated and interesting journal of the events, which remains among his unpublished papers.

In 1789, Mr. Rigby was admitted a member of the London Corporation of Surgeons, and of the London Medical Society.

The natural desire of gradually retiring from the incessant and constantly-increasing labours of a most laborious and responsible profession, induced Mr. Rigby to think of taking the degree of doctor of medicine, and henceforth of practising as a physician: he, therefore, took his diploma in 1814. But, in order to remove the delicate anxiety of females, whom he had hitherto attended, he consented, in many instances, to continue his services to his old patients, while they were needed in one department; thus demonstrating the same kind attention to the feelings of his connexions, for which his life has been remarkable.

Up to the hour of his last illness, he was employed in a sixth edition of his greatest work, the "Essay on the Uterine Hemorrhage."

His personal habits were simple, frugal, and abstemious: he rose very early, drank no wine; and enjoyed, till within a few days of his death, an almost uninterrupted series of health and vigour. Even so late as ten days before his decease, he performed a journey of sixty miles, after visiting his Norwich patients, in one day, although just relieved from a state of considerable muscular pain, under which he had been suffering for some weeks. In fact, his mind never yielded: he was enthusiastic in the pursuit of good, and unwearied in its exercise.

Dr. Rigby was twice married: first, to Sarah, co-heiress of John Dyball, esq.; and, secondly, to Anne, daughter of W. Palgrave, esq. He has left issue two daughters by the first, and two sons and six daughters by the second lady, who has borne him twelve children. Already his progeny has arrived at the fourth generation. He died in his 74th year.

